# A dual role of HIF1α in regulating osteogenesis–angiogenesis coupling

**DOI:** 10.1186/s13287-022-02742-1

**Published:** 2022-02-05

**Authors:** Jingjing Shao, Shibo Liu, Min Zhang, Shujiang Chen, Shuaiqi Gan, Chenfeng Chen, Wenchuan Chen, Lei Li, Zhimin Zhu

**Affiliations:** 1 State Key Laboratory of Oral Diseases, National Clinical Research Center for Oral Diseases, Department of Prosthodontics, West China Hospital of Stomatology, Sichuan University, No 14, Sec. 3, Renminnan Road, Chengdu, 610041 People’s Republic of China; 2 Department of Oral Prosthodontics, West China Hospital of Stomatology, Sichuan University, Chengdu, Sichuan China; 3 Department of Oral and Maxillofacial Surgery, West China Hospital of Stomatology, Sichuan University, Chengdu, Sichuan China

**Keywords:** HIF1α, p53, ROS, Osteogenesis–angiogenesis coupling, Senile osteoporosis

## Abstract

**Objectives:**

The hypoxia-inducible factor 1-α (HIF1α), a key molecule in mediating bone-vessel crosstalk, has been considered a promising target for treating osteoporosis caused by gonadal hormones. However, senile osteoporosis, with accumulated senescent cells in aged bone, has a distinct pathogenesis. The study aimed at revealing the unknown role of HIF1α in aged bone, thus broadening its practical application in senile osteoporosis.

**Materials and methods:**

Femurs and tibias were collected from untreated mice of various ages (2 months old, 10 months old, 18 months old) and treated mice (2 months old, 18 months old) underwent 4-w gavage of 2-methoxyestradiol (a kind of HIF1α inhibitor). Bone-vessel phenotypes were observed by microfil infusion, micro-CT and HE staining. Markers of senescence, osteogenesis, angiogenesis, oxidative stress and expression of HIF1α were detected by senescence β-galactosidase staining, qRT-PCR, western blot and immunostaining, respectively. Furthermore, bone mesenchymal stem cells from young mice (YBMSCs) and aged mice (ABMSCs) were transfected by knockout siRNA and overexpression plasmid of HIF1α. Senescence β-galactosidase staining, Cell Counting Kit-8, transwell assay, alkaline phosphatase staining, alizarin red-S staining and angiogenesis tests were utilized to assess the biological properties of two cell types. Then, Pifithrin-α and Nutlin-3a were adopted to intervene p53 of the two cells. Finally, H_2_O_2_ on YBMSCs and NAC on ABMSCs were exploited to change their status of oxidative stress to do a deeper detection.

**Results:**

Senescent phenotypes, impaired osteogenesis–angiogenesis coupling and increased HIF1α were observed in aged bone and ABMSCs. However, 2-methoxyestradiol improved bone-vessel metabolism of aged mice while damaged that of young mice. Mechanically, HIF1α showed opposed effects in regulating the cell migration and osteogenesis–angiogenesis coupling of YBMSCs and ABMSCs, but no remarked effect on the proliferation of either cell type. Pifithrin-α upregulated the osteogenic and angiogenic markers of HIF1α-siRNA-transfected YBMSCs, and Nutlin-3a alleviated those of HIF1α-siRNA-transfected ABMSCs. The HIF1α-p53 relationship was negative in YBMSCs and NAC-treated ABMSCs, but positive in ABMSCs and H_2_O_2_-treated YBMSCs.

**Conclusion:**

The dual role of HIF1α in osteogenesis–angiogenesis coupling may depend on the ROS-mediated HIF1α-p53 relationship. New awareness about HIF1α will be conducive to its future application in senile osteoporosis.

**Supplementary Information:**

The online version contains supplementary material available at 10.1186/s13287-022-02742-1.

## Introduction

Bone is a hyper-vascularized organ based on the coupling relationship between osteogenesis and angiogenesis [1]. The general maintenance of the bone-vessel niche depends on oxygen and nutrients transported by vascular cells and proangiogenic factors secreted by bone lineage cells. The aging process may disrupt osteogenesis–angiogenesis coupling, leading to senile osteoporosis. A pioneering study discovered that various cell types isolated from bone marrow of old mice, comprising but not limited to osteoblast progenitors, osteoblasts and osteocytes, expressed higher levels of senescence markers than their young counterparts [2]. Bone mesenchymal stem cell (BMSC) and osteoblastic cell senescence induced by DNA damage or telomere erosion using a gene knockout mouse model was verified to lead to damaged osteogenesis, enhanced osteoclastogenesis and osteoporosis genesis [3]. Moreover, the BMSC-mediated proangiogenic potential declines with age and osteoporosis subsequently occurs [4, 5]. Thus, a better target for improving the osteogenesis and angiogenesis of senescent BMSCs is essential for treating senile osteoporosis.

In addition to the typical morphological changes, resulting in a flat and enlarged shape, senescent biomarkers, including senescence-associated-β-galactosidase (SA-β-gal), p53, p21, p16, etc., arise in senescent cells. The notion of p53-dependent initiation and maintenance of senescence have been gradually confirmed [6, 7]. p53 activates p21 and many other pro-senescence targets involved in regulating cellular senescence and age-related diseases, including senile osteoporosis [8, 9]. Moreover, reactive oxidative stress (ROS), produced during disorganized homeostasis of cell metabolism, has been listed as an important trigger for cellular senescence and aging [10]. Although the cause–effect relationship between ROS and p53 is complex and controversial, the ROS/p53 pathway was shown to be an important mediator in the senescence and differentiation of BMSCs [10–12]. Specifically, the accumulated ROS in aged individuals mediated p53 activation that, in turn, skewed the redox equilibrium system toward a pro-oxidative potential and senile osteoporosis.

The key role of hypoxia-inducible factor-1 (HIF-1), an αβ heterodimeric transcription factor, in angiogenesis–osteogenesis coupling was initially revealed [13–15]. Unlike constitutively expressed HIF1β, HIF1α is an adaptive protein modulated by biomechanical and proinflammatory signals during bone regeneration. Increased bone mass and abundant skeletal vasculature were observed in mice overexpressing HIF1α in osteoblasts and enhanced osteogenesis was found in HIF1α-transfected BMSCs [16, 17]. Notably, different aging tissues show various changes in HIF-1α expression [18, 19]. Reduced HIF1α expression was reported in the aging brain, kidney, liver and heart, while increased HIF1α expression was found in aging mucosal tissues. However, few studies have examined its changes in senescent bone, much less how its functional mechanism varies during the aging process.

Herein, we investigated the effect of HIF1α on osteogenesis and angiogenesis at different ages and the intermediary role of ROS and p53 in regulating the effect of HIF1α. In this way, a comprehensive conception of HIF1α-regulated aging-related bone metabolism would be constructed, and practical applications of HIF1α in senile osteoporosis could be promoted.

## Materials and methods

### Animal preparation and processing

#### Animal preparation

C57BL/6 J male mice of various ages (2 months old, 10 months old, 18 months old) were purchased from Dossy Experimental Animals Company (Chengdu, China). Femurs and tibias were collected and processed according to the guidelines of the subsequent experiments.

#### Animal processing

2-month-old and 18-month-old mice were administered 75 mg/kg 2-methoxyestradiol (2-ME2, a HIF1α inhibitor, MCE, USA) by daily gavage for 4 w. 2-ME2 was prepared at 15 mg/ml in a 0.5% methylcellulose solution (MERCK, USA). Then, the femurs and tibias were isolated for later analysis.

### Microfil perfusion and Micro-CT (microcomputed tomography) assay

#### Microfil perfusion

The mice were sacrificed and successively perfused with 100 U/ml heparin solution, 4% paraformaldehyde and microfil reagent (MV-117, Flow Tech Inc, USA) [20]. After 4 °C overnight, the femurs and tibias were dissected, fixed for 48-h in 4% paraformaldehyde and decalcified for 3-w in 15% EDTA.

#### Micro-CT (microcomputed tomography) assay

The distal femurs and mesial tibias (undecalcified and decalcified) were scanned with micro-CT using a resolution of 10 μm. 200 slices under the growth plates of the femurs and tibias were defined as the region of interest to evaluate the bone and vascular microstructure. Different parameters, such as BV/TV (bone volume/tissue volume), Tb. Th (trabecular thickness), Tb. N (trabecular number), Tb. Sp (trabecular separation), vascular volume, vascular number, vascular thickness and vascular separation, were then calculated to compare the variance among the groups.

### Histological observation and immunostaining

#### Senescence β-galactosidase (SA-β-gal) staining

Frozen sections of the femurs and tibias were stained with the Senescence β-galactosidase Staining Kit (Solarbio, China).

#### HE staining and immunostaining

After dewaxing and rehydration, paraffin sections were subjected to hematoxylin–eosin staining (Solarbio, China). For immunostaining, a series of procedures, including antigen retrieval, hydrogen peroxide blocking and BSA blocking, were carried out, followed by primary antibody (listed in Additional file 3: Table S1) incubation overnight and secondary antibody (listed in Additional file 3: Table S1) incubation the next day. Immunochemical staining was accomplished through subsequent coloration and hematoxylin counterstaining, while immunofluorescence staining was performed with DAPI (Solarbio, China).

### Cell isolation and culture

#### BMSCs

Bilateral femurs and tibias were obtained from young (2-month-old) and aged (18-month-old) C57BL/6 J male mice, respectively. Young BMSCs (YBMSCs) and aged BMSCs (ABMSCs) were then flushed out from the bone marrow and cultured with complete α-MEM (Gibco, USA) supplemented with 10% fetal bovine serum (FBS, Gibco, USA) and 1% penicillin/streptomycin (Gibco, USA). The third passage of cells was used for the subsequent experiments.

#### Human umbilical vein endothelial cells (HUVECs)

HUVECs were provided by State Key Laboratory of Oral Diseases, West China Hospital of Stomatology, Sichuan University, and were cultured in low-glucose DMEM (Gibco, USA) supplemented with 10% FBS and 1% penicillin/streptomycin.

All of the cells were incubated in an environment with appropriate humidity at 37 °C and 5% CO_2_.

### Knockout and overexpression transfection of HIF1α

After cell adherence to 6-well plates, YBMSCs and ABMSCs were transfected with 100 pmol HIF1α siRNA/500 ng pCMV-HIF1α (overexpression plasmid) (Gene Pharma, China) per well using transfection medium that included 5‰ Endofectin (a transfection reagent, Gene Pharma, China). Scramble siRNA and empty plasmid were used as corresponding blank controls. The transfection process lasted for 8 h, and then, the cells were cultured with complete media for at least another 48 h.

### Cell phenotypic observation

#### SA-β-gal staining

YBMSCs and ABMSCs were cultured in 6-well plates and stained with the Senescence β-galactosidase Staining Kit (Solarbio, China).

#### Cell proliferation assay

Briefly, after cell adherence to 96-well plates, YBMSCs and ABMSCs were transfected with HIF1α siRNA or HIF1α overexpression plasmid and the corresponding empty controls. Cell Counting Kit-8 (CCK-8, Dojindo, Japan) was used to detect the effect of HIF1α on the proliferation of BMSCs at 24 h and 48 h.

#### Cell migration assay

Transwell assay was used to detect the migration potential of BMSCs. After adherence to the top chamber, the cells were transfected with siRNA or overexpression plasmid. 8 h later, the medium of the top chamber was changed to FBS-free medium, and the bottom chamber was filled with complete medium. After another 24 h, the cells were fixed with 4% paraformaldehyde and stained with 0.5% crystalline violet dye solution (Solarbio, China). The cells in the top chamber were removed, and the cells in the bottom chamber were observed and counted under an optical microscope.

#### Osteogenic differentiation assay

BMSCs were induced by osteogenesis induction media after transfection. Alkaline phosphatase (ALP) staining and alizarin red-S (ARS) staining were carried out by a BCIP/NBT Alkaline Phosphatase Color Development Kit (Beyotime, China) and alizarin red-S solution (Solarbio, China), respectively.

#### Immunofluorescence staining in vitro

BMSCs were fixed with 4% paraformaldehyde, permeabilized with 0.5% Triton X-100 (Solarbio, China) and then incubated with primary antibody (listed in Additional file 3: Table S1) overnight and secondary antibody the next day. DAPI and TRITC-phalloidin (Solarbio, China) were used to stain the nucleus and cytoskeleton, respectively.

#### Measurement and intervene of ROS

To intervene in the status of ROS in different groups, YBMSCs were pretreated with 200 μM H_2_O_2_ (Sigma, USA) for 24 h, and ABMSCs were pretreated with 500 μM NAC (Beyotime, China) for 30 min. Then, the cells were incubated in FBS-free medium containing 10 μM 2′,7′‐dichlorofluorescin diacetate (H_2_DCF‐DA, Sigma‐Aldrich) for 40 min. The absorbance was read using a microplate reader at the appropriate wavelength.

### qRT-PCR (quantitative real-time PCR)

Total RNA was extracted from bone samples and cell lysates with TRIzol reagent and reverse transcribed into cDNA using the PrimeScript RT reagent kit (Takara, Japan). One microliter of cDNA was amplified by 2 × SYBR Green qPCR Master Mix (Bimake, China) and specific primers (listed in Additional file 4: Table S2) following the manufacturer's instructions. Gene expression was calculated for at least three independent experiments and normalized to the expression level of β-actin.

### Western blot

Protein lysates were obtained from bone samples and cell lysates using RIPA lysis buffer (Solarbio, China). After electrophoresis, the membrane was blocked with 5% BSA, and the membranes were incubated with primary antibodies (listed in Additional file 3: Table S1) overnight at 4 °C. The next day, the membranes were incubated with the corresponding secondary antibodies (listed in Additional file 3: Table S1), and an ECL kit (US Everbright Inc., China) was used.

### Angiogenesis assay

HUVECs were treated with conditioned medium from different groups of BMSCs (CM-BMSCs). Four kinds of angiogenesis tests were used to investigate the indirect angiogenic effect of BMSCs regulated by HIF1α.

#### ELISA assay

The CM-BMSCs were collected and centrifuged to obtain liquid supernatant. Then, the concentration of VEGF-A protein in the supernatant was detected by the VEGF-A ELISA kit (Cloud-Clone, China).

#### Wound healing assay

A 200 μl pipette tip was used to scratch plates with confluent cells. Before and 24 h after the medium was changed to CM-BMSCs, the scratch areas were photographed and wound healing percentages (areas of migrating cells/total scratch areas) were measured by ImageJ software (Additional file 1: Fig. S1).

#### Transwell assay

The procedure was carried out as in Cell migration assay.

#### Tube formation assay

Cells were resuspended in CM-BMSCs and seeded onto 24-well plates precoated with Matrigel (Corning, USA). After incubation for 6 h, the cells were fixed with 4% paraformaldehyde, incubated with 0.5% Triton X-100 and stained with TRITC-phalloidin and DAPI. The tubular structure was photographed with an inverted fluorescence microscope. Subsequent quantitative analysis was performed by ImageJ software.

## Statistics analysis

Data were calculated from at least three independent experiments and were presented as the means ± S.D. Comparisons among groups were made with one-way or two-way ANOVA. *p* < 0.05 means a significant difference.

## Results

### Phenotypic characteristics of bone and vessels declined with age in mice

**Fig. 1:**
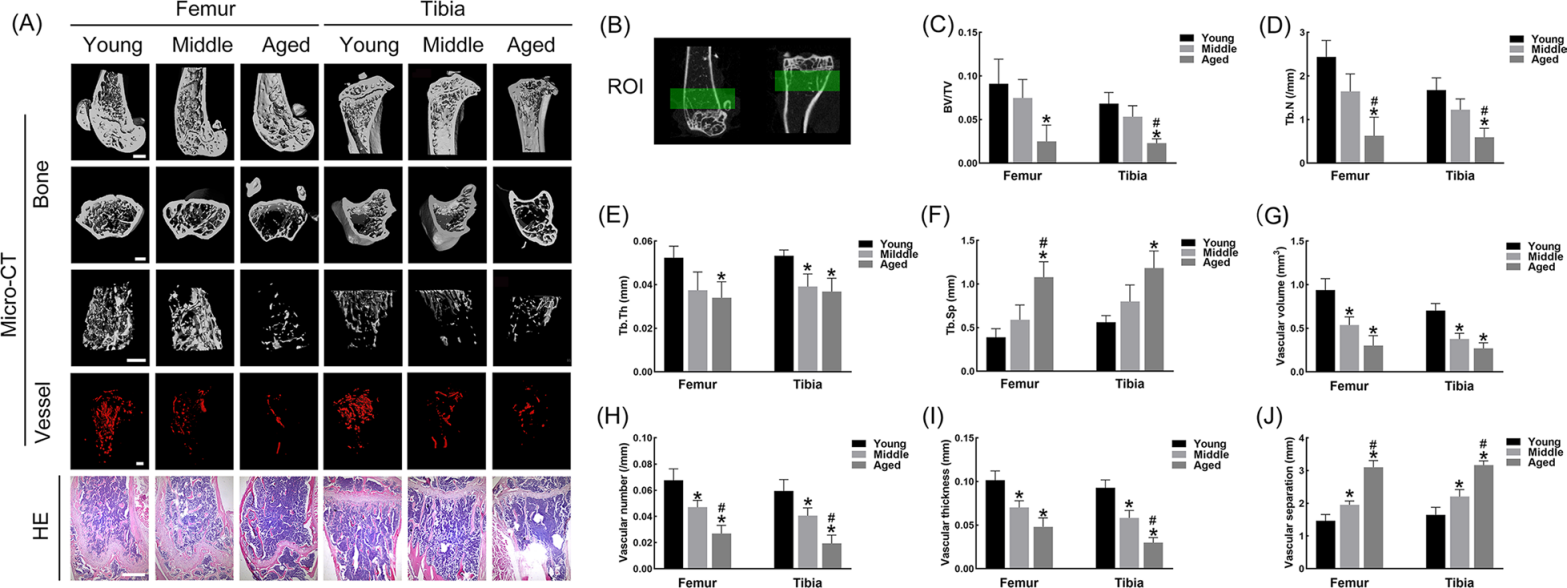
Bone-vessel coupling declined with age in the femurs and tibias of mice. **A** Representative 3D-reconstructed images by Micro-CT and morphological observation by HE staining. Bar = 500 μM. **B** Region of interest (ROI) for quantitative analysis. **C**–**J** BV/TV, Tb.N, Tb,Th, Tb.Sp, vascular volume, vascular number, vascular thickness and vascular separation among groups. *p < 0.05 versus Young; ^#^*p* < 0.05 versus Middle

From the multi-perspective 3D reconstructed images, both femurs and tibias showed sparse and thin trends in the trabecular bone and vessels with age (Fig. 1A). HE staining visually displayed the most sparsely distributed trabecular bone in the aged mice (Fig. 1A). Relative parameters (BV/TV, Tb. N, Tb. Th, Tb. Sp, vascular volume, vascular number, vascular thickness and vascular separation) also demonstrated age-related changes quantitatively (Fig. 1C–J).

### Osteogenesis and angiogenesis in vivo are passively related to HIF1α and p53

**Fig. 2:**
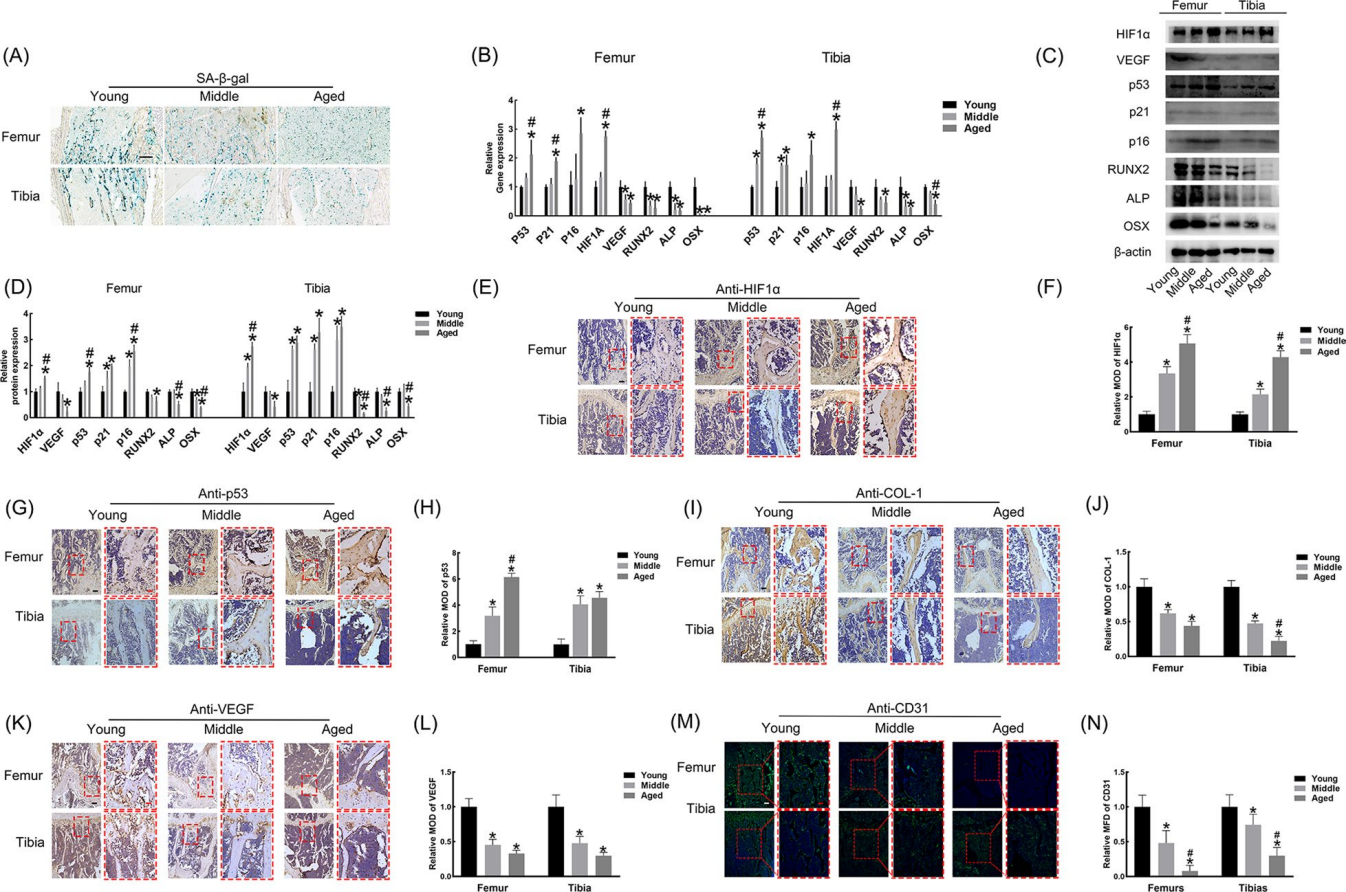
Age-related degenerated osteogenesis and angiogenesis were related to upregulated HIF1α and p53. **A** SA-β-gal staining in femurs and tibias. Bar = 250 μM. **B** Relative gene expression analysis of femurs and tibias. **C**, **D** Relative protein expression analysis of femurs and tibias. **E**–**N** Immunostaining analysis of HIF1α, p53, COL-1, VEGF and CD31 in femurs and tibias. Black/white bar = 100 μM; red bar = 25 Μm. MOD (mean optical densities) = Integral optical density/Area and MFD (mean fluorescence density) = Integral fluorescence density/Area. *p < 0.05 versus Young; ^#^*p* < 0.05 versus Middle

The number of β-gal-positive cells in aged bone was significantly higher than that in young bone (Fig. 2A). Meanwhile, osteogenic markers (RUNX2, ALP and OSX) and angiogenic marker (VEGF) all decreased gradually with age, while senescent markers (p53, p21 and p16) and HIF1α showed an upward trend (Fig. 2B–D). For immunochemistry analysis, the HIF1α and p53 immunostaining intensities continuously increased with age (Fig. 2E–H). In addition, osteogenic marker (COL-1) and angiogenic markers (CD31 and VEGF) manifested a downward tendency with age (Fig. 2I–N).

### Inhibition of HIF1α could improve the bone-vessel metabolism of aged mice and worsen that of young mice

**Fig. 3:**
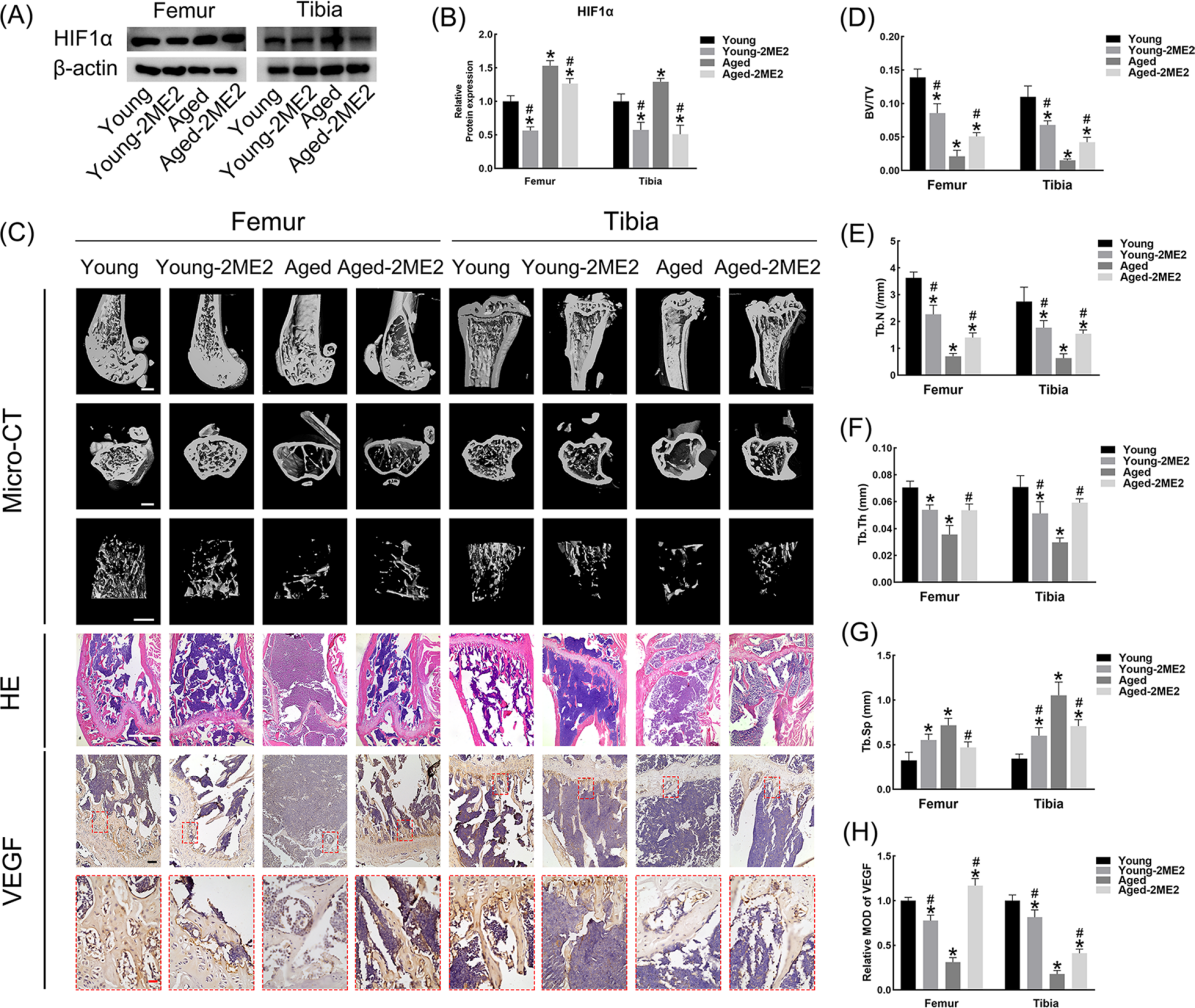
2-ME2 alleviated the bone-vessel metabolism of aged mice, but impaired that of young mice. **A**, **B** Western blot analysis for HIF1α expression in femurs and tibias. **C** Representative 3D-reconstructed images by micro-CT, morphological observation by HE staining and immunostaining analysis of VEGF in femurs and tibias. White bar = 500 μM; Black bar = 100 μM; Red bar = 25 μM. **D**–**G** BV/TV, Tb.N, Tb,Th, Tb.Sp among groups. **H** Relative MOD of VEGF by immunostaining. *p < 0.05 versus Young; ^#^*p* < 0.05 versus Aged

Protein analysis showed that 4 w gavage of 2-ME2 successively inhibited the HIF1α expression in the bone of either young or aged mice (Fig. 3A, B). Based on the phenotypic observation, 2-ME2 remarkably made the trabecular bone sparser and thinner in the young mice. However, the bones of the aged mice that underwent 2-ME2 treatment were denser and thicker than the untreated group (Fig. 3C–G). In addition, VEGF was strongly expressed in the bone of the young group but was significantly decreased in that of the young-2ME2 group. Unlike the young mice, the administration of 2-ME2 to aged mice dramatically ameliorated the depressed VEGF expression level in the bone (Fig. 3C, H).

### ABMSCs showed degenerative properties accompanied by the upregulated HIF1α and p53

**Fig. 4:**
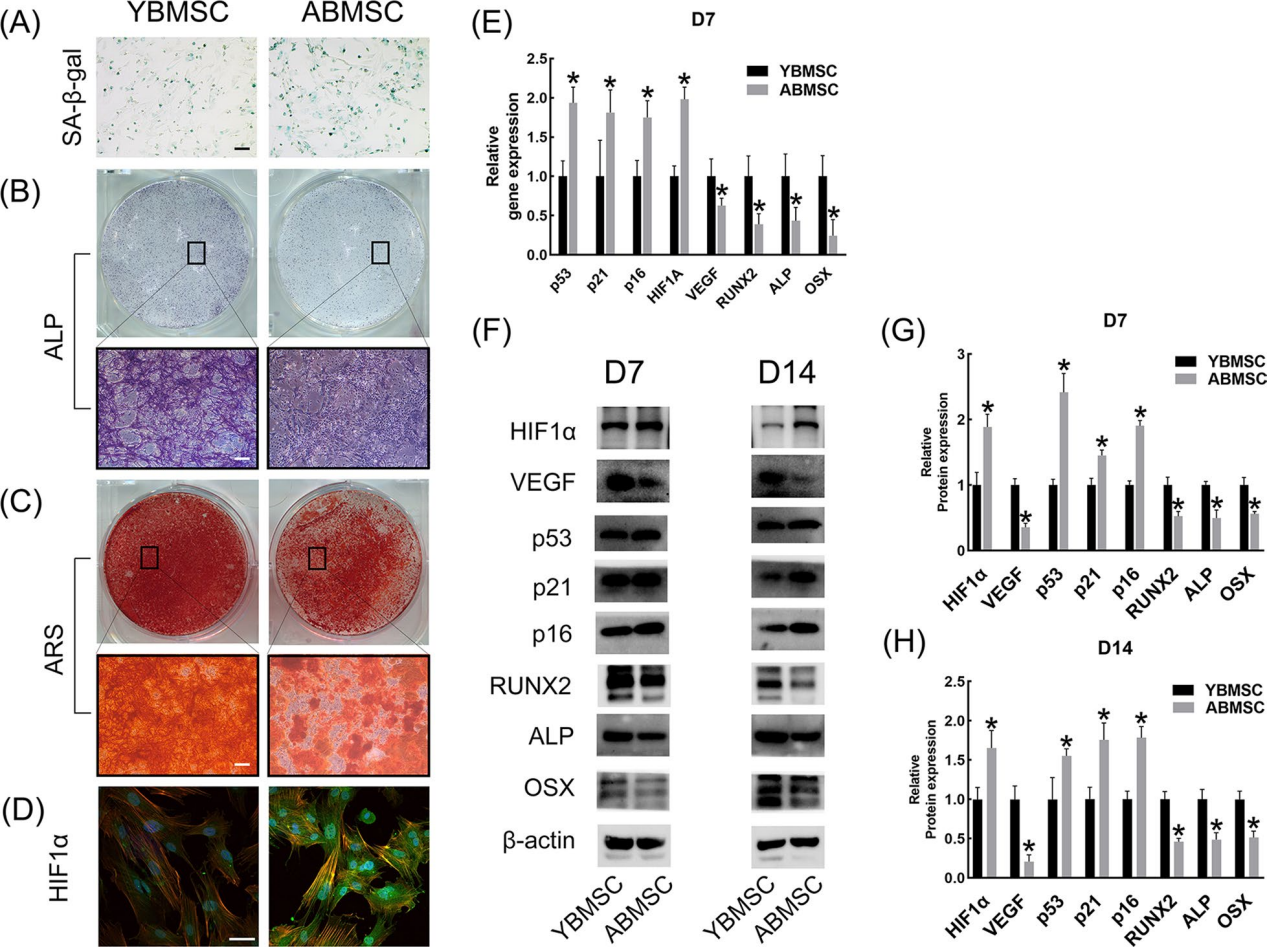
ABMSCs showed degenerative properties and upregulated HIF1α. **A** SA-β-gal staining in YBMSCs and ABMSCs. Bar = 100 μM. **B**, **C** ALP staining after 7-d osteogenic induction and ARS staining after 21-d osteogenic induction. Bar = 200 μM. **D** Immunostaining of HIF1α in YBMSCs and ABMSCs. Bar = 50 μM. **E** Relative gene expression of YBMSCs and ABMSCs after 7-d osteogenic induction. **F**–**H** Relative protein expression of YBMSCs and ABMSCs after 7-d and 14-d osteogenic induction, respectively. *p < 0.05 versus YBMSC

The percentage of β-gal-positive cells in the ABMSCs was remarkably higher than that in the YBMSCs (Fig. 4A). ALP and ARS staining qualitatively illustrated the inhibited osteogenic differential potential of ABMSCs (Fig. 4B, C). Differentially expressed HIF1α, senescent markers and osteogenic and angiogenic markers in the two cell types were also detected at both the gene and protein levels. Unlike in the YBMSCs, HIF1α and senescent markers were upregulated in the ABMSCs, while the remaining indicators were downregulated (Fig. 4D–4H, Additional file 2: Fig. S2).

### HIF1α showed opposite effects on the migration ability of YBMSCs and ABMSCs but no obvious effect on the proliferation of either cell type

**Fig. 5:**
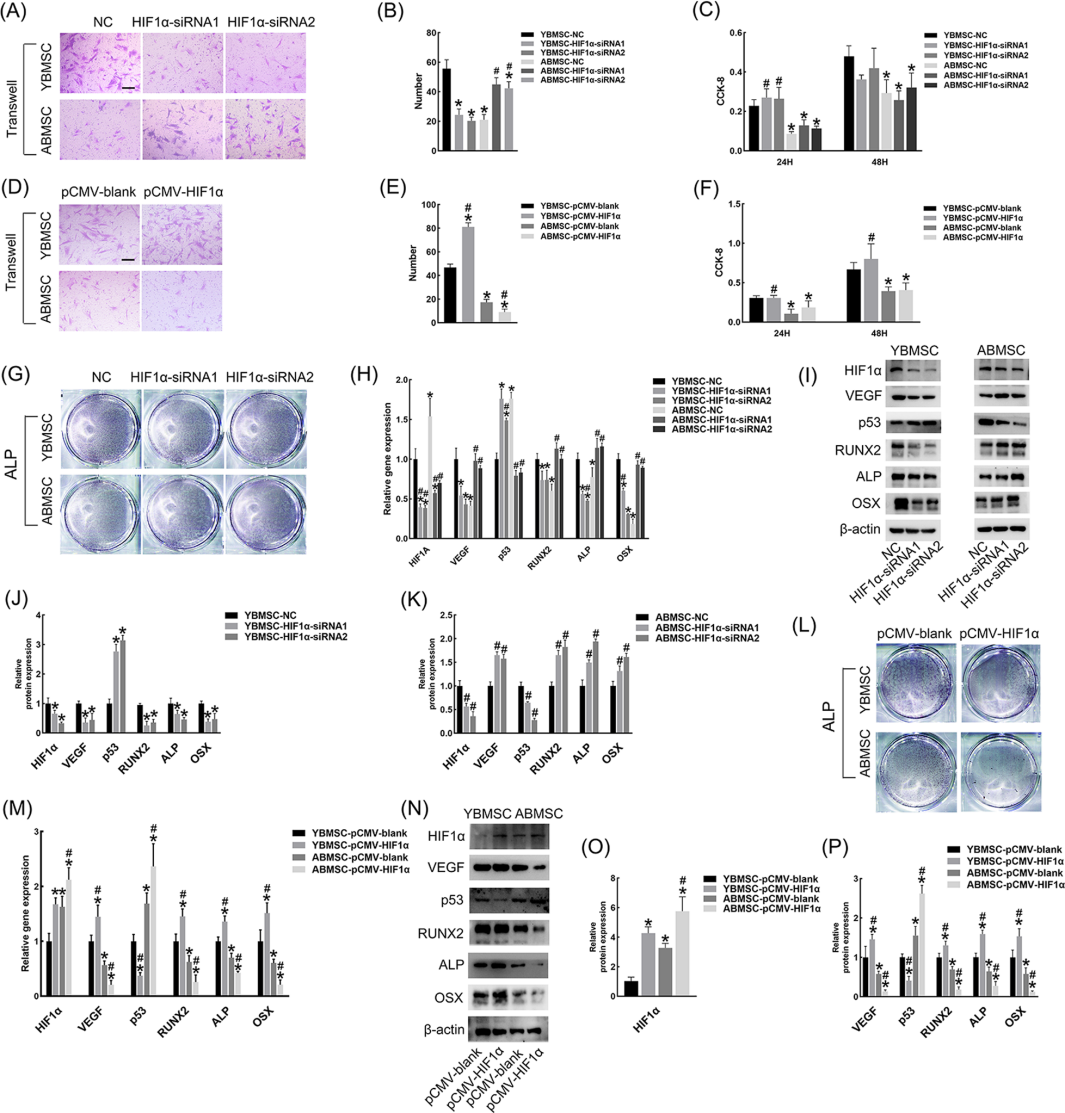
HIF1α showed opposite effects on the migration ability and osteogenesis of YBMSCs and ABMSCs, but no obvious effect on the cell proliferation. **A**, **B**, **D**, **E** Transwell assay of BMSCs interfered with HIF1α-siRNA or pCMV-HIF1α. Bar = 100 μM. **C**, **F** CCK-8 assay at 24 h and 48 h. **G,L**, ALP staining after 4-d osteogenic induction. **H**, **M** Relative gene expression of transfected BMSCs after 3-d osteogenic induction. **I**–**K**, **N**–**P** Relative protein expression of transfected BMSCs after 3-d osteogenic induction. *p < 0.05 versus YBMSC-NC or YBMSC-pCMV-blank; ^#^*p* < 0.05 versus ABMSC-NC or ABMSC- pCMV-blank

Knocking out HIF1α reduced the migratory activity of YBMSCs, and HIF1α overexpression facilitated those of YBMSCs, while ABMSCs reacted in the opposite way (Fig. 5A, B, D, E). However, there was no significant difference in cell proliferation when comparing the knockout group and overexpression group with the corresponding control groups (Fig. 5C, F).

### HIF1α played a dual role in regulating the osteogenesis–angiogenesis coupling of YBMSCs and ABMSCs

ALP staining after 4 d of osteogenic induction showed that the suppressed osteogenesis of ABMSCs was alleviated after transfection, while that of YBMSCs was decreased (Fig. 5G). At the gene and protein levels, the downregulated osteogenic and angiogenic markers of ABMSCs, such as ALP, RUNX2, OSX and VEGF, recovered to different degrees when the ABMSCs were transfected with HIF1α siRNA, while those of YBMSCs were decreased (Fig. 5H–K). The opposite effect of the HIF1α overexpression plasmid on BMSCs is shown in Fig. 5L–P.

**Fig. 6:**
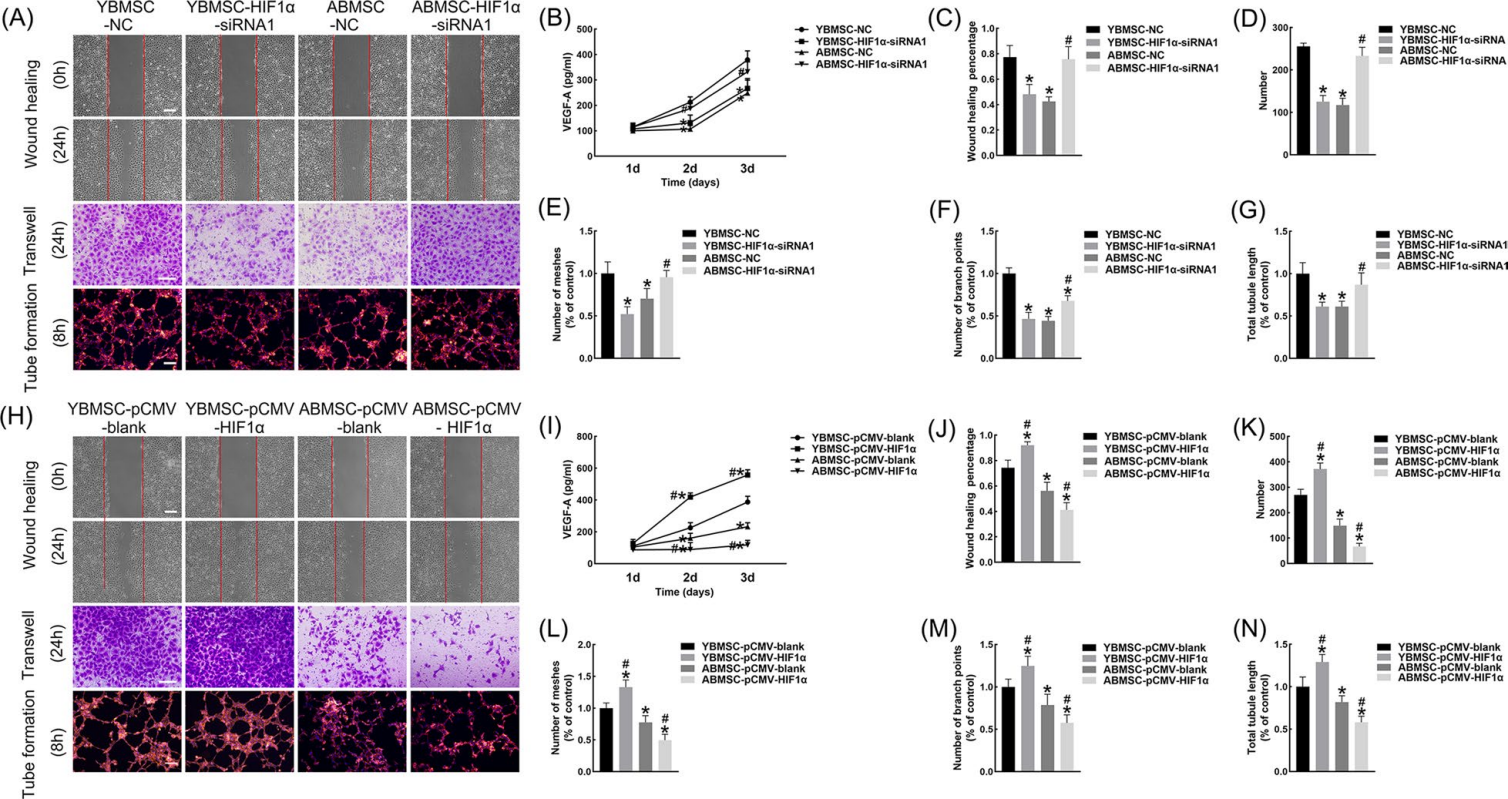
HIF1α facilitated the indirect angiogenic ability of ABMSCs and inhibited that of YBMSCs. **A**, **C**–**G**, **H**, **J**–**N** Wound healing assay, transwell assay and tube formation assay of HUVECs after 24-h CM-BMSCs treatment. Bar = 100 μM. **B**, **I** Concentration of VEGF-A in different groups by Elisa. CM, conditional medium collected from BMSCs. *p < 0.05 versus YBMSC-NC or YBMSC-pCMV-blank; ^#^*p* < 0.05 versus ABMSC-NC or ABMSC- pCMV-blank

The indirect angiogenic effect of BMSCs mediated by VEGF was reflected in the migration and tube formation ability of HUVECs. The highest and lowest levels of VEGF were detected in the CM-YBMSC-NC and CM-ABMSC-NC groups, respectively (Fig. 6B). The transfection of HIF1α-siRNA inhibited the secretion of VEGF by YBMSCs and promoted that of ABMSCs (Fig. 6B). In accordance with the ELISA results, CM-ABMSC-HIF1α-siRNA had a better ability to induce the migration and tube formation of HUVECs than CM-ABMSC-NC, while the effect of CM-YBMSC-HIF1α-siRNA on HUVECs was remarkably worse than that of CM-YBMSC-NC (Fig. 6A, C–G). In contrast, HIF1α overexpression in BMSCs facilitated the osteogenesis–angiogenesis coupling of YBMSCs and impaired that of ABMSCs (Fig. 6H–N).

### p53 mediated the HIF1α-regulated osteogenesis and angiogenesis

**Fig. 7:**
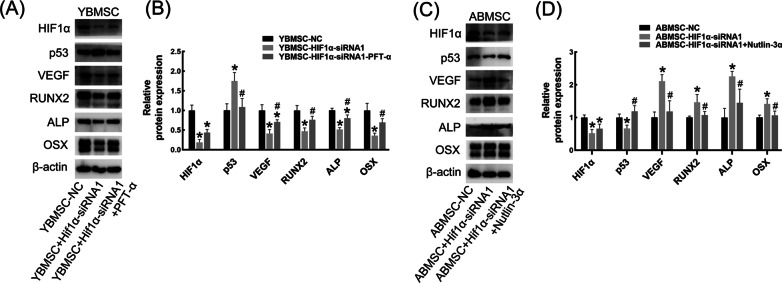
P53 mediated the effect of HIF1α on YBMSCs and ABMSCs. **A**, **B** Quantitative protein analysis for YBMSCs interfered with HIF1α-siRNA and PFT-α. **C**, **D** Quantitative protein analysis for ABMSCs interfered with HIF1α-siRNA and Nutlin-3α. *p < 0.05 versus YBMSC-NC or ABMSC-NC; ^#^*p* < 0.05 versus YBMSC-HIF1α-siRNA or ABMSC- HIF1α-siRNA

Pifithrin-α (PFT-α, 10 μM), a p53 inhibitor, and Nutlin-3a (10 μM), a p53 activator, were introduced to verify the role of p53 in HIF1α-regulated osteogenesis–angiogenesis coupling. The level of p53 expression in the YBMSC-HIF1α-siRNA-PFT-α group was effectively decreased by PFT-α, while Nutlin-3a significantly increased the low level of p53 expression in the ABMSC-HIF1α-siRNA group (Fig. 7). In addition, the downregulation of osteogenic and angiogenic markers in the YBMSC-HIF1α-siRNA group was obviously relieved by PFT-α, and the upregulation of relevant markers in the ABMSC-HIF1α-siRNA group was reduced by Nutlin-3a (Fig. 7).

### Effect of HIF1α on p53 was concerned with the status of oxidative stress

**Fig. 8:**
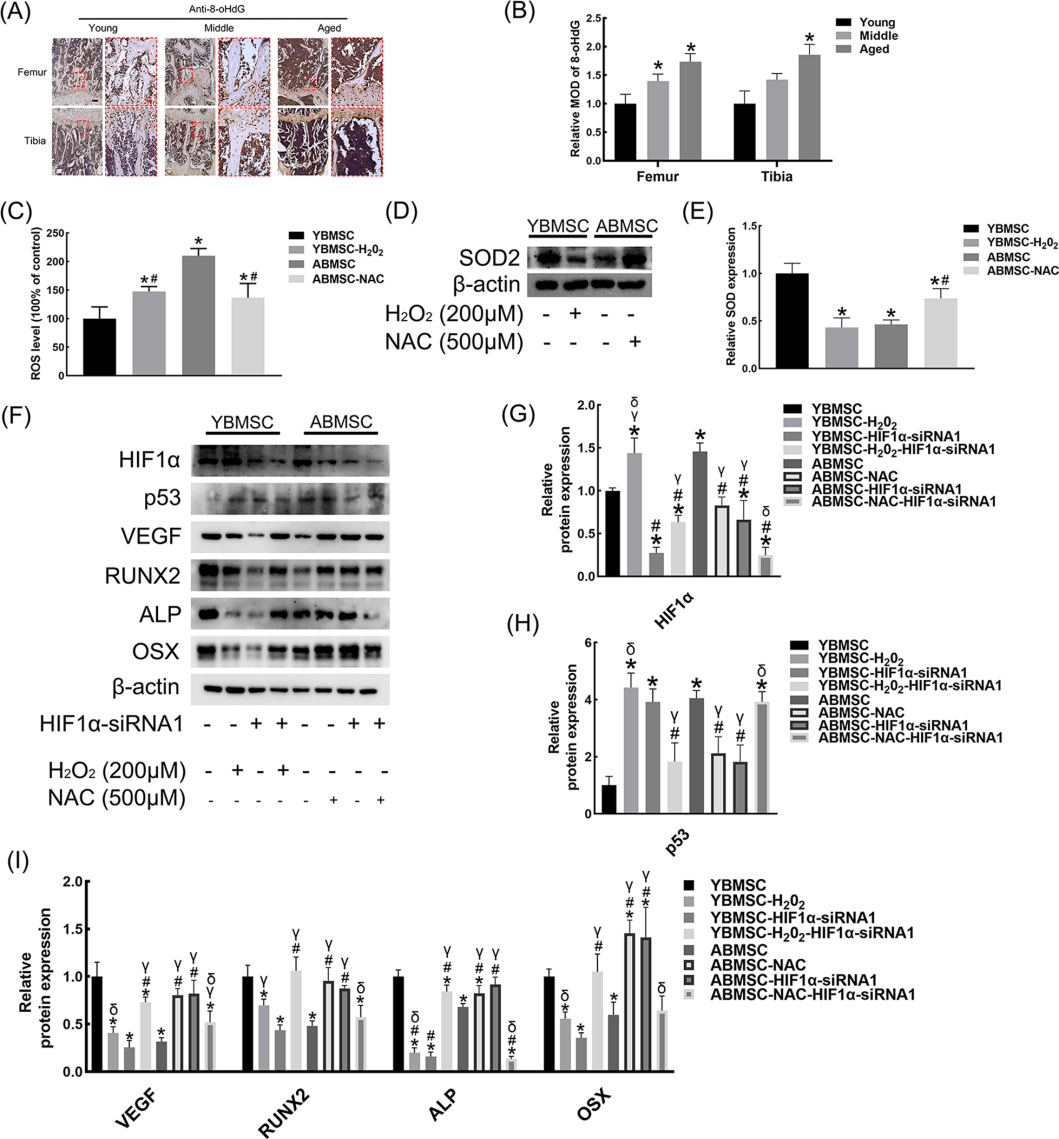
ROS regulated the HIF1α-p53 axis. **A**, **B** 8-oHdG staining of femurs and tibias. Black bar = 100 μM; red bar = 25 μM. **C** Quantitative analysis of relative ROS level in YBMSCs and ABMSCs. **D**, **E** Relative SOD_2_ expression of H_2_O_2_-treated YBMSCs and NAC-treated ABMSCs. **F** Western blot analysis for HIF1α-siRNA-transfected H_2_O_2_-treated YBMSCs and NAC-treated ABMSCs. **G** Quantitative analysis of HIF1α. **H**, **I** Quantitative analysis of p53, VEGF, RUNX2, ALP and OSX. *p < 0.05 versus YBMSC; ^#^*p* < 0.05 versus ABMSC; γ*p* < 0.05 versus YBMSC-HIF1α-siRNA; δ*p* < 0.05 versus ABMSC-HIF1α-siRNA

8-oHdG (an oxidative stress marker) accumulated with age in the long bone (Fig. 8A, B). Significantly different ROS levels between YBMSCs and ABMSCs were also discovered. H_2_O_2_ and NAC successfully increased the level of ROS in YBMSCs and decreased that in ABMSCs (Fig. 8C). In addition, the change in Superoxide dismutase-2 (SOD_2,_ an important antioxidation marker) was in contrast to that of ROS (Fig. 8D, E). Subsequent western blot assays showed a bilateral HIF1α-p53 relationship under different oxidative stress conditions. In YBMSCs or H_2_O_2_-induced ABMSCs, HIF1α knockout promoted p53 accumulation and impaired osteogenesis–angiogenesis coupling (Fig. 8F–I). In addition, in ABMSCs or NAC-induced YBMSCs, the p53 expression level and osteogenesis–angiogenesis coupling markers were reversed by HIF1α-siRNA (Fig. 8F–I).

## Discussion

**Fig. 9:**
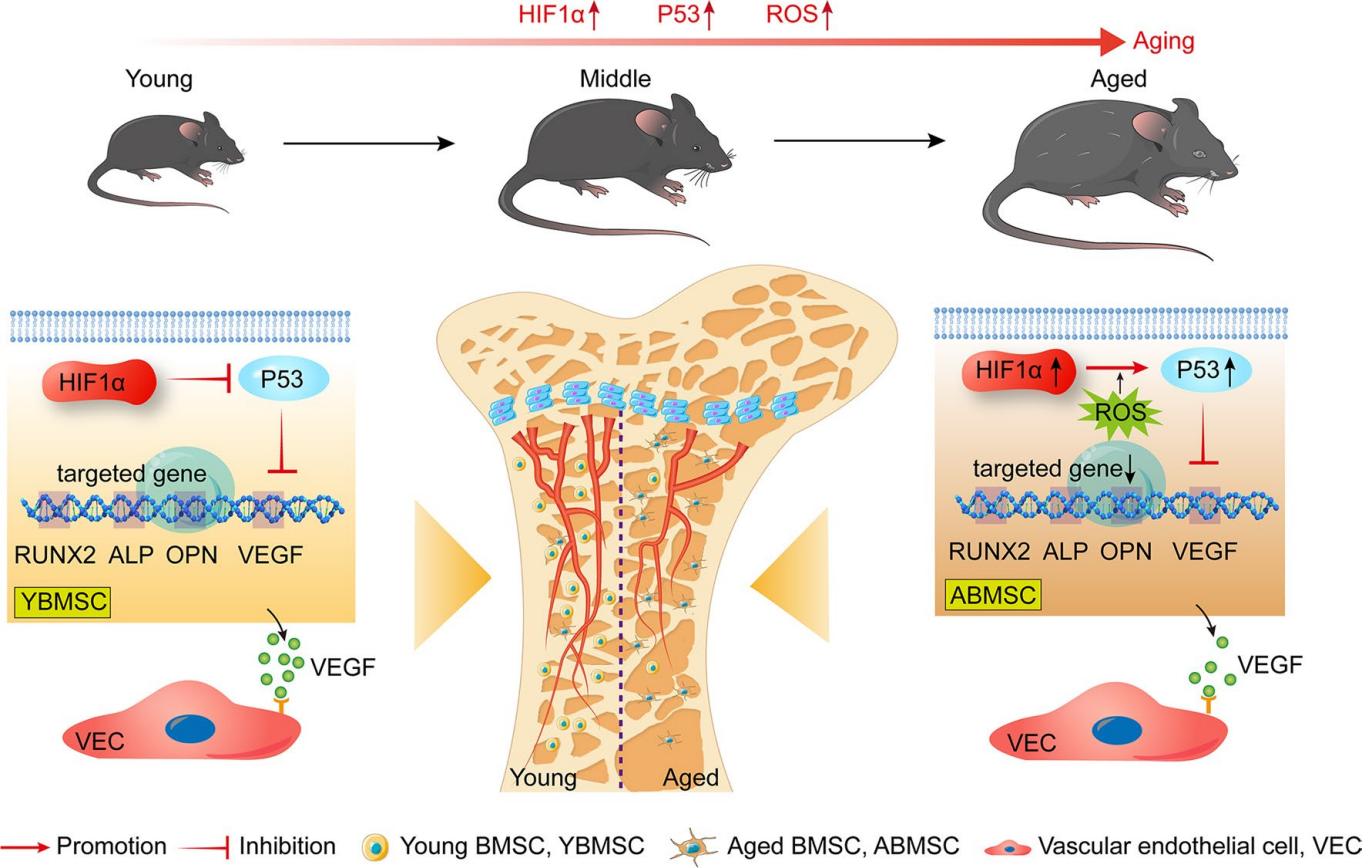
A sketch map of HIF1α -regulated osteogenesis–angiogenesis coupling

The features of senile osteoporosis, including decreased bone mass, degenerative bone quality and impaired vessel formation, are being validated by multiple animal models [21]. A concurrent hotspot in research for alleviating degenerative osteogenesis–angiogenesis coupling is the investigation of gene therapy based on the underlying pathogenic mechanisms [22, 23]. HIF1α is one of the essential factors in mediating bone-vessel crosstalk. Interestingly, we found that HIF1α increased with age in the long bone and that inhibition of HIF1α in bone could improve damaged osteogenesis and angiogenesis. Moreover, HIF1α was demonstrated to play a dual role in regulating osteogenesis–angiogenesis coupling, and the effect was age-related and closely associated with ROS-mediated HIF1α/p53 axis (Fig. 9).

Physiologically, hypoxia is believed to be critical for activating the HIF1α signaling pathway, which initiates angiogenesis and impinges on the occurrence of endochondral ossification in the long bone [17]. A dramatic result was reported by Clemens et al., in which mice overexpressing or lacking HIF1α in mature osteoblasts developed dense or thin skeleton phenotypes, respectively, and this effect could only be realized in the presence of blood vessels. Moreover, HIF1α is activated during the process of bone repair. With genetic editing or pharmacological methods used to change HIF1α expression, multiple pathological bone metabolism diseases are attenuated, including oral periapical lesions, osteoporosis caused by estrogen or androgen deficiency, pathologic bone fractures and cranial defects [1, 24–28]. However, age, an important factor affecting bone metabolism, has not been fully considered in HIF1α-related bone regulation. It was recently proposed that chronic hypoxia may develop in the physiologically aging retina and that age-related macular degeneration is induced by the subsequent increased activity of HIF1α- and HIF1α-targeted genes [29]. However, in this study, upregulated HIF1α and downregulated osteogenesis and angiogenesis were discovered in aged bone and ABMSCs. Thin and less vascularized bone in aging mice may impair oxygen delivery to the osteoblast-vascular niche and then constantly activate HIF1α in BMSCs. However, an inhibitor of HIF1α successfully improved damaged bone-vessel metabolism in aged mice.

In the case of the opposite variation between HIF1α and osteogenesis–angiogenesis coupled with age, it is intriguing to further unravel the role of HIF1α in regulating bone metabolism. Ordered production of ossification and blood vessels constitute bone formation and bone remodeling [30, 31]. At the molecular level, multiple osteogenic and angiogenic factors secreted by autocrine and/or paracrine mechanisms are involved in linking BMSCs and vascular endothelial cells and impacting their biological properties, such as recruitment, proliferation and differentiation [32]. VEGF, an important factor mediating osteogenesis–angiogenesis coupling, has already been reported as a key reference marker to evaluate the angiogenic potential of BMSCs [33–35]; hence, the angiogenesis of CM-BMSC-treated HUVECs was detected to further test the effect of BMSCs on osteogenesis–angiogenesis coupling. Here, we found that YBMSCs performed identically to these previous studies, which reported a positive correlation between HIF1α and the bone-vascular phenotype, while ABMSCs had the opposite phenotype.

In fact, senescence status may determine the various reactions of BMSCs to HIF1α. There is no sex difference in the rate of bone loss in 5–10 years after the menopausal phase, suggesting that the common pathogenesis for senile osteoporosis, in both females and males, is the aging process of bone cells and the bone marrow microenvironment [36]. In this study, normally aging mice (18 months old) were utilized to study senile osteoporosis due to their similarity to humans in terms of age-related changes in bone [37, 38]. More positive reactions for SA-β-gal staining and higher expression of p53, p21 and p16 were discovered in vitro and in vivo, signifying an increase in senescent cells in aged tissue. The accumulation of senescent cells impairs skeletal homeostasis and executes age-related bone loss [36]. Mechanistically, the initiation and maintenance of senescence are both regulated by p53. In replicative senescence, p53 is activated by the progressive erosion of telomeres to elicit genomic instability, which leads to a plethora of aging-related phenotypes, including osteoporosis [39, 40]. Mice with p53 deletion systemically or specifically in BMSCs showed a remarkable increase in bone density and trabecular thickness compared with control littermates [41]. As observed in this research, promotion or suppression of p53 directly intervened in the effect of HIF1α on osteogenesis and angiogenesis, which further hinted at the possibility that p53 worked as a downstream signal of HIF1α in its effect on bone metabolism.

Notably, p53 was negatively related to HIF1α in YBMSCs and was positively related to HIF1α in ABMSCs. The regulating effect of a third factor was implied in the opposite HIF1α-p53 link between the two cells. Redox homeostasis maintains stemness and prevents cell senescence [42]. In view of the intensified 8-OhdG staining in vivo and destroyed redox equilibrium in vitro, it was apparent that ROS accumulated in the aged bone and ABMSCs. In addition, additional alterations in ROS reversed the HIF1α-p53 link. It has been conclusively demonstrated that p53 responds to HIF1α differently according to the degree of hypoxic status [43]. Under mild hypoxia, partially activated HIF1α was insufficient to inhibit the Mdm2-dependent degradation of p53, thus leading to the downregulation of p53. Nevertheless, under moderate and even severe hypoxia, HIF1α became fully activated and promoted the stabilization of p53. Analogous to hypoxia, it could be speculated that the degree of oxidative stress may also modulate the relationship between HIF1α and p53. Increasing amounts of evidence has suggested that ROS can mediate the stabilization, transactivation, transcription and translation of HIF1α by regulating signaling molecules such as ERK and PI3K/AKT [44]. From the moderate ROS in YBMSCs to the severe ROS in ABMSCs, the stabilized and transactivated HIF1α gradually became sufficient to retard the degeneration of p53, thus explaining the converted regulatory effect of HIF1α on p53.

## Conclusions

Cell senescence, a concomitant factor of age, complicates the properties of BMSCs and challenges the treatment of senile osteoporosis. Here, we initially proved that HIF1α regulated the osteogenesis–angiogenesis coupling of long bone bidirectionally and that this function was mediated through the ROS-HIF1α/p53 axis. With the above premise, a new idea for treating senile osteoporosis is revealed. However, a secure clinical application of HIF1α depends on a deeper investigation into the protein, requiring more study in the future.

## Supplementary Information

**Additional file 1: Fig. S1.** A diagram of quantitative analysis for wound healing assay.

**Additional file 2: Fig. S2.** Separated images of HIF1α-staining in YBMSCs and ABMSCs.

**Additional file 3: Table S1.** Information of antibody used in the experiment.

**Additional file 4: Table S2.** Information of primer sequences used in the experiment.

## Data Availability

All data generated or analyzed during this study are included in this published article.

## Abbreviations

SA-β-gal: Senescence-associated-β-galactosidase
ROS: Reactive oxidative stress
HIF-1: Hypoxia-inducible factor-1
2-ME2: 2-Methoxyestradiol
Micro-CT: Microcomputed tomography
BV/TV: Bone volume/tissue volume
Tb. Th: Trabecular thickness
Tb. N: Trabecular number
Tb. Sp: Trabecular separation
BMSCs: Bone mesenchymal stem cell
FBS: Fetal bovine serum
HUVEC: Human umbilical vein endothelial cell
CCK-8: Cell Counting Kit-8
ALP: Alkaline phosphatase
ARS: Alizarin red-S
H_2_DCF‐DA: 2′,7′-Dichlorofluorescin diacetate
PFT-α: Pifithrin-α
SOD_2_: Superoxide dismutase-2
