# Supplementary figures and images for: A dual role of HIF1α in regulating osteogenesis–angiogenesis coupling
Source: Stem Cell Res Ther. 2022 Feb 5;13:59. doi: 10.1186/s13287-022-02742-1 (PMC8818171; doi:10.1186/s13287-022-02742-1)

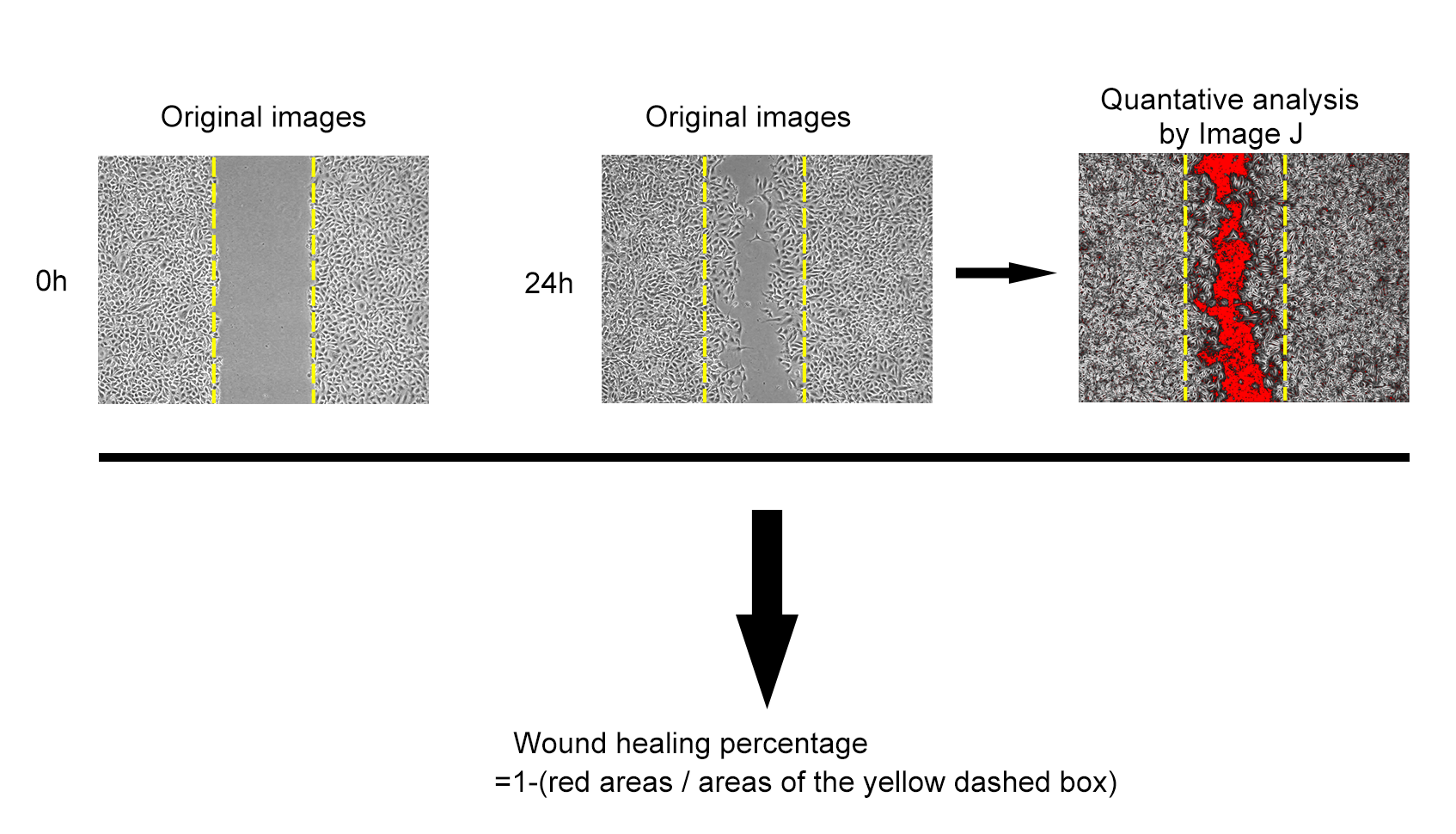

Supplement: Supplementary file 1 — Additional file 1: Fig. S1. A diagram of quantitative analysis for wound healing assay. [file 13287_2022_2742_MOESM1_ESM.tif]

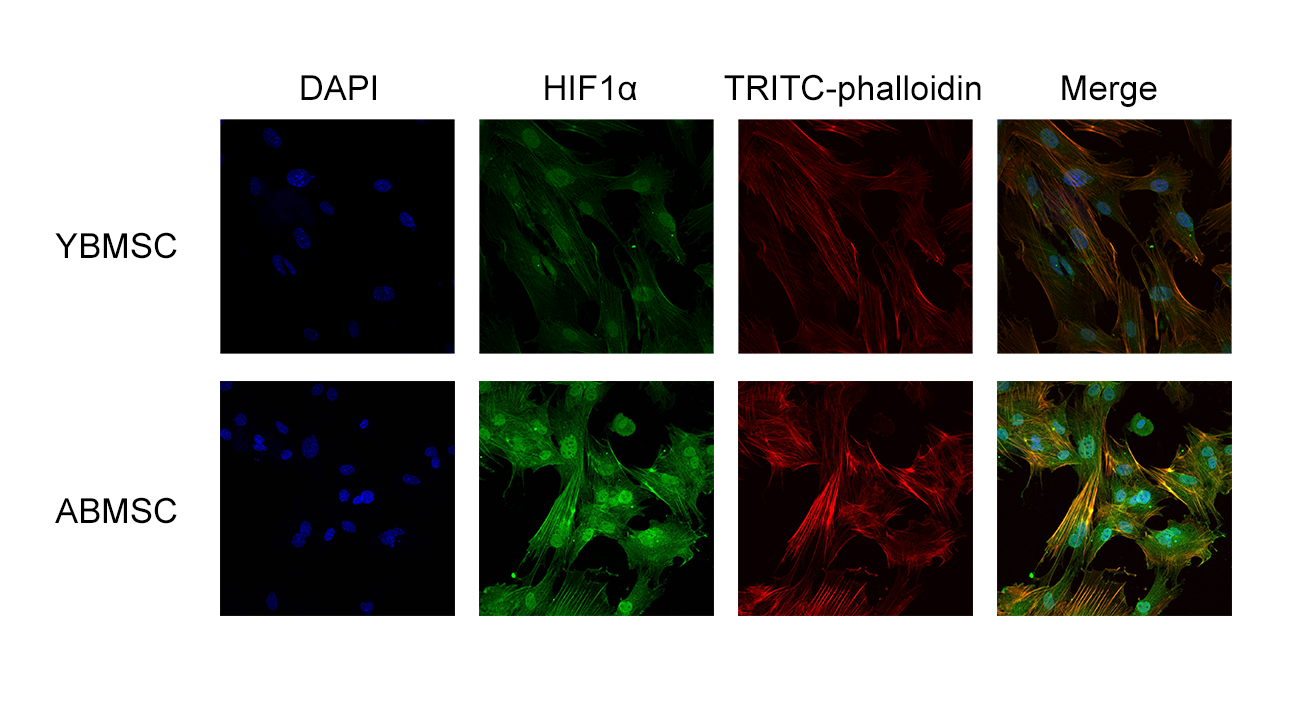

Supplement: Supplementary file 2 — Additional file 2: Fig. S2. Separated images of HIF1α-staining in YBMSCs and ABMSCs. [file 13287_2022_2742_MOESM2_ESM.tif]
