# Supplementary material for: A dual role of HIF1α in regulating osteogenesis–angiogenesis coupling
Source: Stem Cell Res Ther. 2022 Feb 5;13:59. doi: 10.1186/s13287-022-02742-1 (PMC8818171; doi:10.1186/s13287-022-02742-1)
Supplement: Supplementary file 3 — Additional file 3: Table S1. Information of antibody used in the experiment. [file 13287_2022_2742_MOESM3_ESM.docx]

| Antibody Brand Product code Application proportion |
| --- |
| HIF1α Affinity AF1009 WB 1:1000; IHC/IF 1:200 |
| p53 Abcam ab131442 WB 1:1000; IHC 1:200 |
| p21 Abcam ab188224 WB 1:1000 |
| p16 Abcam ab51243 WB 1:1000 |
| VEGF HuaBio ET1604-28 WB 1:1000; IHC 1:200 |
| RUNX2 CST 12556S WB 1:1000 |
| ALP HuaBio ET1601-21 WB 1:1000 |
| OSX Abcam ab209484 WB 1:1000 |
| SOD2 Abcam ab68155 WB 1:1000 |
| COL-1 Abcam ab254113 IHC 1:200 |
| CD31 HuaBio ER31219 IF 1:200 |
| 8-ohdG Abcam ab48508 IHC 1:200 |
| β-actin CST 4970T WB 1:1000 |
| Anti-rabbit IgG,  HRP-linked Antibody CST 7074S WB 1:2000 |
| FITC Conjugated Goat anti-Rabbit  IgG Goat Polyclonal Antibody HuaBio HA1004 IF 1:200 |
| Note: WB: Western Blot; IHC: Immunohistochemistry; IF: Immunofluorescence |
