# Supplementary material for: A dual role of HIF1α in regulating osteogenesis–angiogenesis coupling
Source: Stem Cell Res Ther. 2022 Feb 5;13:59. doi: 10.1186/s13287-022-02742-1 (PMC8818171; doi:10.1186/s13287-022-02742-1)
Supplement: Supplementary file 4 — Additional file 4: Table S2. Information of primer sequences used in the experiment. [file 13287_2022_2742_MOESM4_ESM.docx]

| Gene Forward Primer (5’-3’) Reverse Primer (5’-3’) |
| --- |
| HIF1α ACCTTCATCGGAAACTCCAAAG CTGTTAGGCTGGGAAAAGTTAGG |
| p53 CTCTCCCCCGCAAAAGAAAAA CGGAACATCTCGAAGCGTTTA |
| p21 CCTGGTGATGTCCGACCTG CCATGAGCGCATCGCAATC |
| p16 CGCAGGTTCTTGGTCACTGT TGTTCACGAAAGCCAGAGCG |
| VEGF CTGCCGTCCGATTGAGACC CCCCTCCTTGTACCACTGTC |
| RUNX2 TTCAACGATCTGAGATTTGTGGG GGATGAGGAATGCGCCCTA |
| ALP CCAACTCTTTTGTGCCAGAGA GGCTACATTGGTGTTGAGCTTTT |
| OSX ATGGCGTCCTCTCTGCTTG TGAAAGGTCAGCGTATGGCTT |
| β-actin GGCTGTATTCCCCTCCATCG CCAGTTGGTAACAATGCCATGT |
